# Decayed, missing, and restored teeth in patients with Neurofibromatosis Type 1

**DOI:** 10.4317/jced.54561

**Published:** 2018-02-01

**Authors:** Reinhard E. Friedrich, Anika Reul

**Affiliations:** 1 Prof. Dr. med. Dr. med. dent, Department of Oral and Craniomaxillofacial Surgery; 2 Senior registrar, Department of Prosthodontics, Eppendorf University Hospital, University of Hamburg, Hamburg, Germany

## Abstract

**Background:**

NF1 is a relatively frequently occurring autosomal dominant inherited disease. There are conflicting reports about oral health status in NF1. The aim of this study was to analyze the dental status of patients with neurofibromatosis type 1 (NF1).

**Material and Methods:**

Radiographs of 179 patients with NF1 were analyzed for decayed, missing, and filled teeth (DMFT) in a cross-sectional, retrospective study. The results were compared to age- and sex-matched controls of individuals not affected by NF1. The NF1 group was differentiated for facial tumor type and localization.

**Results:**

Missing teeth were more frequently registered in the NF1 group. On the other hand, decayed teeth were more frequent in the reference group. However, these findings had to be interpreted with caution, because the type and localization of the facial tumor affected the measured values.

**Conclusions:**

Dental health in terms of DMFT differed between NF1 patients and the control group. The presented results indicate the need for special care in dentistry in NF1 patients in order to preserve dental health, particularly in individuals affected with certain types of facial tumors.

** Key words:**DMFT index, neurofibromatosis type 1, plexiform neurofibroma, oral health.

## Introduction

Neurofibromatosis type 1 (NF1) is an autosomal dominant tumor predisposition syndrome (1). About 1:2500 children living at birth are affected with the disease (2-5). The gene locus of NF1 is 17q11.2 (6-10). About every second individual diagnosed as a patient affected by NF1 has no known ancestors who have also suffered from this disease (5). Neurofibroma is a benign nerve sheath tumor and the hallmark of the disease (11). Mutations in Schwann cells are the cause of nerve sheath tumors in NF1 (12). The disease is characterized by a large number of manifestations in different organs and tissues (13,14) and also affects multiple regions of the orofacial system (15-17).

Several craniofacial findings are diagnostic for NF1 during clinical assessment of an individual (5). The retention of teeth, complex tooth deficiencies, and unusual jaw deformities are included in the spectrum of oral manifestations of this entity (18-22). Difficulties in oral surgical procedures and in the maintenance of teeth in patients with NF1 have been reported (23-26). Recently, reports have been published that show either a significantly poorer or better preservation of dental health in patients with NF1 compared to the respective reference population (27,28). These contradictory reports gave reason to evaluate the dental health of our own patients based on the analysis of radiographs of a larger group of patients.

## Material and Methods

Individuals. The basis of this study was the orthopantomograms (OPGs) of patients with NF1. Diagnosis of NF1 was established in every single patient according to current guidelines (5). Radiographs were performed during routine clinical investigation in order to search for dental diseases, odontogenic or disease-associated jaw lesions, and malformations of the jaws known to occur in this entity. This study was approved by the local institutional board of the hospital as a prerequisite for a medical dissertation in dentistry (AR). All patients gave informed consent to the scientific study of x-ray images and evaluation of medical findings. All procedures performed in this study involving human participants were in accordance with the ethical standards of the institutional and/or national research committee and with the 1964 Declaration of Helsinki and its later amendments or comparable ethical standards. Data were anonymized prior to analysis, and the investigators studying the radiographs were blinded for diagnosis, the identity of individuals, and assignment of the single case to a diagnostic group. These investigations of anonymized data don’t require an ethics vote and were performed in accordance with Hamburgisches Gesundheitsdienstgesetz.

The age and sex of every patient and the date of the radiograph were registered for further anonymous data processing. The patients were also evaluated for tumorous manifestations in the facial region. Patients with disseminated cutaneous neurofibromas constituted one group (DCNF). A second group was constituted of all patients affected with facial plexiform neurofibroma (FPNF group). In the latter group, the extension of FPNF roughly correlates to the innervation fields of the trigeminal nerve (29). FPNF can affect a single or numerous branches of the fifth cranial nerve. Therefore, the FPNF group was further subtyped according to the visible facial tumor extension ( Table 1). However, this classification of tumor extension was also supported with the aid of magnetic resonance images, B-scan ultrasound images, and histological diagnoses following surgical procedures, if applicable. FPNF developed in every case unilaterally. In order to identify the impact of tumor localization on dental status, the laterality of FPNF was registered in every case. Females were slightly more affected by FPNF than males (F/M = 35:32).

**Table 1:**
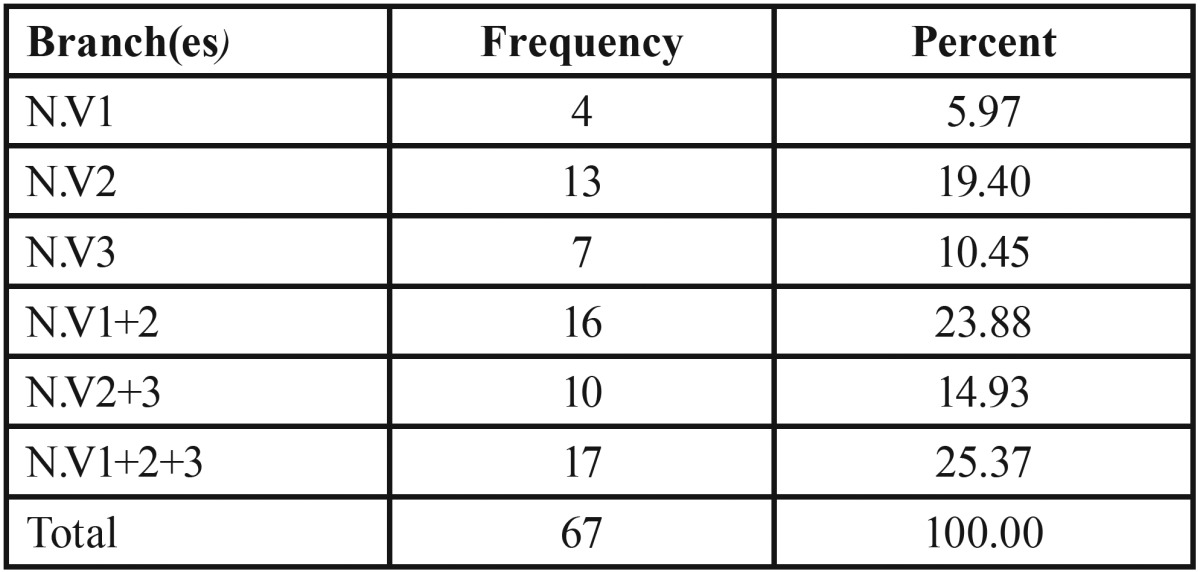
Affected trigeminal nerve branches in patients with facial plexiform neurofibroma (FPNF), n = 67.

NF1 patients with OPGs were excluded from evaluation, which did not represent the entire region of the jaws, as were patients with a history of facial trauma or skeletal surgical procedures in the jaw regions. Furthermore, we excluded three NF1 patients with PNF that originated from the hypoglossal nerve in order to obtain clearly defined NF1 subgroups. A total of 179 patients were included in this study (male: 79, female: 100; mean age: 34.8 years, range: 12 to 68 years). In this study, the development of the tooth should be largely complete. Therefore, we chose as an inclusion criterion of a radiograph that second molars had reached the occlusal plane (22). This study is confined to evaluation of permanent teeth, excluding wisdom teeth. Radiographs of an age- and sex-matched control group (mean age: 34.4 years, range: 12 to 69 years) were derived from the files of the Department of Diagnostic Radiology in Dentistry, University Hospital of Hamburg, and evaluated in the same way as in the examination group.

Diagnostics. All OPGs were of good quality and allowed analysis of tooth pathologies. OPGs of NF1 patients were produced from 1990 to 2008. The majority of radiographs had been performed in the outpatient clinic of the dental or maxillofacial surgery department of the university hospital. However, many patients had their own radiographs for the consultation, either for themselves or their children. These radiographs were archived as films or as digital data storage and were also considered for investigation. Radiographs of the first 15 years of the recruitment period were predominantly archived as films. These radiographs were scanned and digitized for the purpose of this study, as previously described (25). The radiographs of the control group were all taken from the digital archive of the dental clinic. Technical details of the production of these radiographs are described elsewhere in detail (30). Decayed, missing, and filled teeth. A decayed tooth is radiologically defined as radiotranslucency of dental hard tissue. The lesion develops typically on the surface of the tooth and is wedge-shaped. If the dentine is affected by the caries, a more flat spreading of the lesion can be expected. Progressive stages of caries are regularly associated with partial or substantial loss of the tooth shape. This study classifies teeth as either decayed or not decayed. A carious lesion can also be located on a filling edge or crown rim. However, according to the definition of the Decayed-Missing-Filled Teeth (DMFT) index, any case with a decayed tooth in proximity to a dental restoration is defined as a decayed tooth. In these cases, the tooth is assessed as carious and not as filled. The distinction between caries and cavity lining is based on the geometric fit of the latter and its defined limiting lines. However, both superficial occlusal caries and very thin occlusal tooth linings may not be visible on OPGs. This limitation of radiological examination is valid for both groups. A tooth is termed “filled” if the x-ray image images a radiopaque structure that is suitable in extent and shape to restore the tooth shape. A tooth is classified as missing if the radiograph does not show the expected radiopaque structure. The individual qualitative findings were collected for each tooth of the permanent dentition and finally recorded as an individual sum score. The assessment and evaluation of the findings follow the guidelines of the DMFT index in the clinical evaluation of caries prevalence (31,32).

Statistics. For the mean value comparison of the two groups, t-test was used for unrelated samples. Intra-individual side comparison of the dental findings is based on the t-test for connected samples. A *p*-value <0.05 was considered significant. 

## Results

DMFT index. All missing, decayed, and filled or otherwise restored permanent teeth were collected for the DMFT index. Third molars were excluded from evaluation. The mean DMFT index of the patient group was 15.15 and of the control group 12.82 (*p* = 0.004). Within the group of NF1 patients, the mean DMFT index of the FPNF group was 14.60 and of the DCNF group 15.48 (*p* = 0.481).

The lowest mean DMFT index (12.71) was collected in the subgroup with NF1 patients affected solely in the mandibular nerve. Higher DMFT values were obtained in FPNF patients affected in several branches of the trigeminal nerve (first and second trigeminal branch: 15.94; second and third trigeminal branch: 18.80; all branches: 13.35) ( Table 2). However, comparison of mean DMFT indices of FPNF subgroups with the mean DMFT index of the reference group revealed statistically significant differences only for patients simultaneously affected in the maxillary and mandibular nerves (*p* = 0.013, t-test).

**Table 2:**
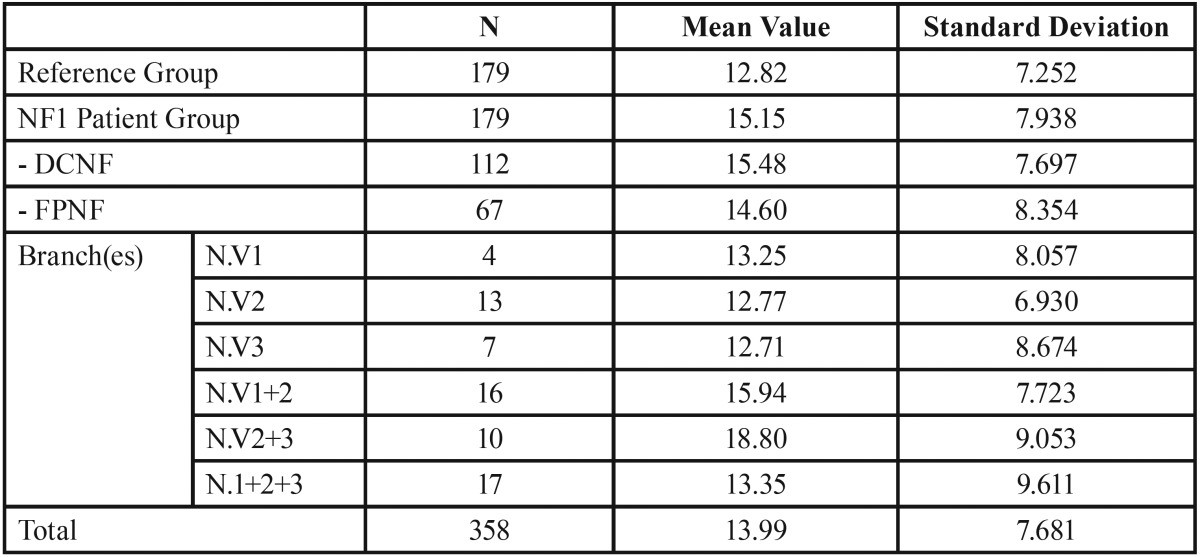
DMFT index in reference and patient groups. Patient group is further specified for trigeminal nerve branch affected by facial plexiform neurofibroma (DCNF = disseminated cutaneous neurofibroma; FPNF = facial plexiform neurofibroma; N. V = nervus trigeminus; numbers 1 and 3 refer to trigeminal nerve branch(es) affected by PNF: 1 = ophthalmic branch, 2 = maxillary branch, 3 = mandibular branch).

Decayed teeth. Comparison of groups revealed a statistically significant higher mean value in the reference group (2.44) compared to the NF1 group (1.59) (*p* = 0.002). Subtyping of the NF1 group disclosed a very low value of decayed teeth in the DCNF group (1.13). On the other hand, the FPNF group (2.34) did not differ from the reference group with respect to this finding (2.44). Further specification of the FPNF group was performed with respect to the localization of the facial tumor to body side and consecutive collection of the number of decayed teeth. This intra-individual comparison of the number of decayed teeth revealed lower mean values on the affected side (0.97 vs. 1.37, *p* = 0.03).

Missing teeth. The number of missing teeth is significantly higher in patients with NF1 compared to the reference group (5.49 vs. 2.49, *p* < 0.001). Within the NF1 group, the number of missing teeth did not differ between patients with disseminated tumors and those with the plexiform type (DCNF: 5.51, FPNF: 5.46). Further investigation of the FPNF group showed that the distribution pattern of the trigeminal tumors has an influence on the number of missing teeth. For instance, patients affected in the first trigeminal branch showed a low mean number of missing teeth (0.5), whereas this value was substantially higher (7.8) in those simultaneously affected in both the maxillary and the mandibular branch. In general, all FPNF groups showed higher mean values of missing teeth compared to the reference group (excepting individuals with exclusive ophthalmic branch affection). However, this difference was statistically significant only in the group of individuals with hemifacial PNF (*p* = 0.004) and cutaneous neurofibroma (*p* < 0.001, t-test), ( Table 3- Table 5).

**Table 3:**
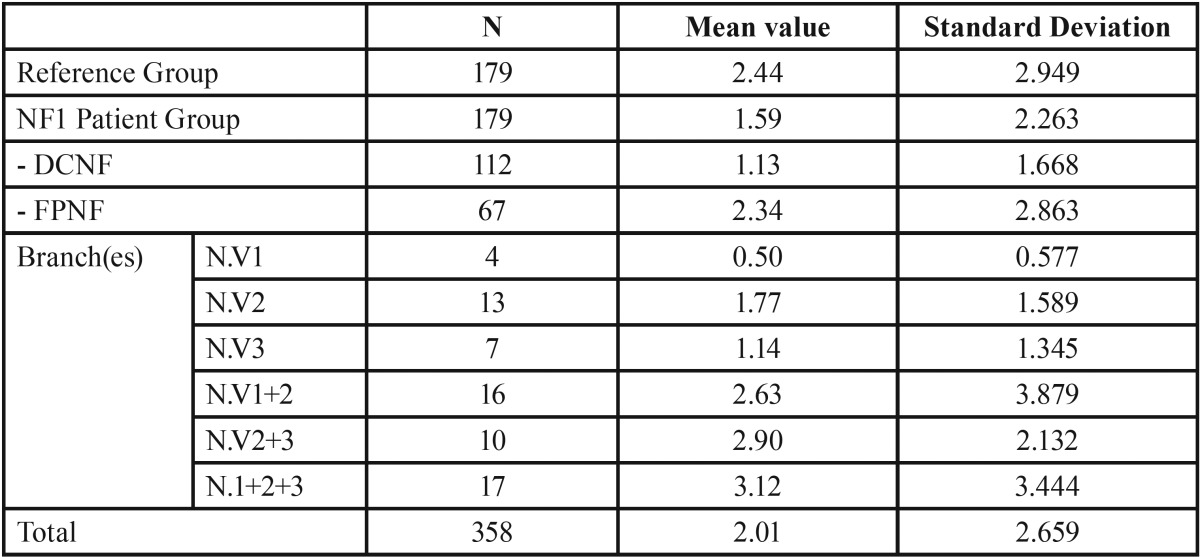
Number of carious lesions in reference group and patient group. Patient group is further specified for trigeminal nerve branch affected by facial plexiform neurofibroma (DCNF = disseminated cutaneous neurofibroma; FPNF = facial plexiform neurofibroma; N. V = nervus trigeminus; numbers 1 and 3 refer to trigeminal nerve branch(es) affected by PNF: 1 = ophthalmic branch, 2 = maxillary branch, 3 = mandibular branch).

**Table 4:**
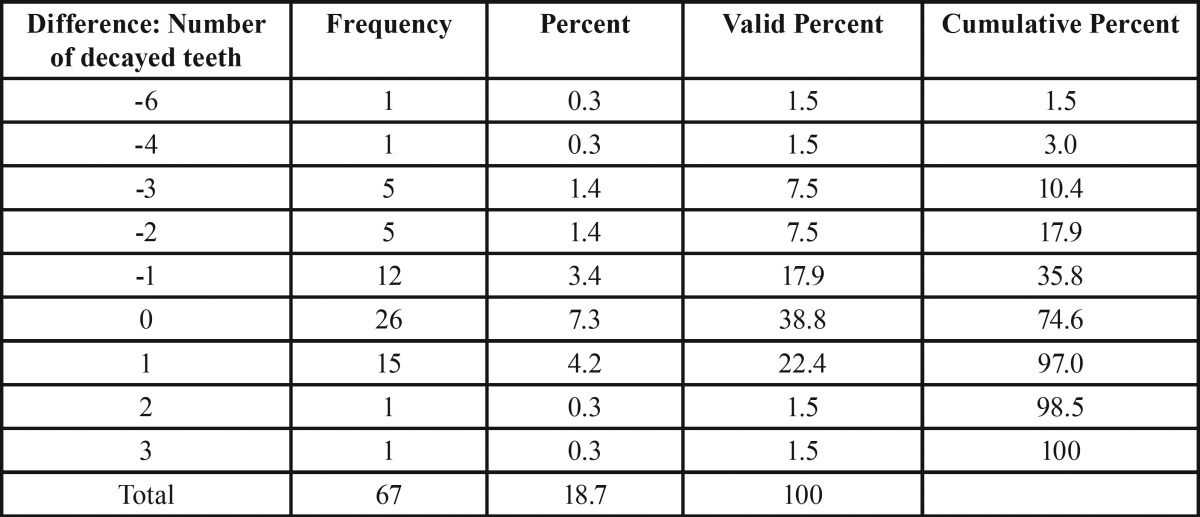
Differences between number of carious teeth per body side with respect to the side affected by facial plexiform neurofibroma (FPNF group, n = 67).

**Table 5:**
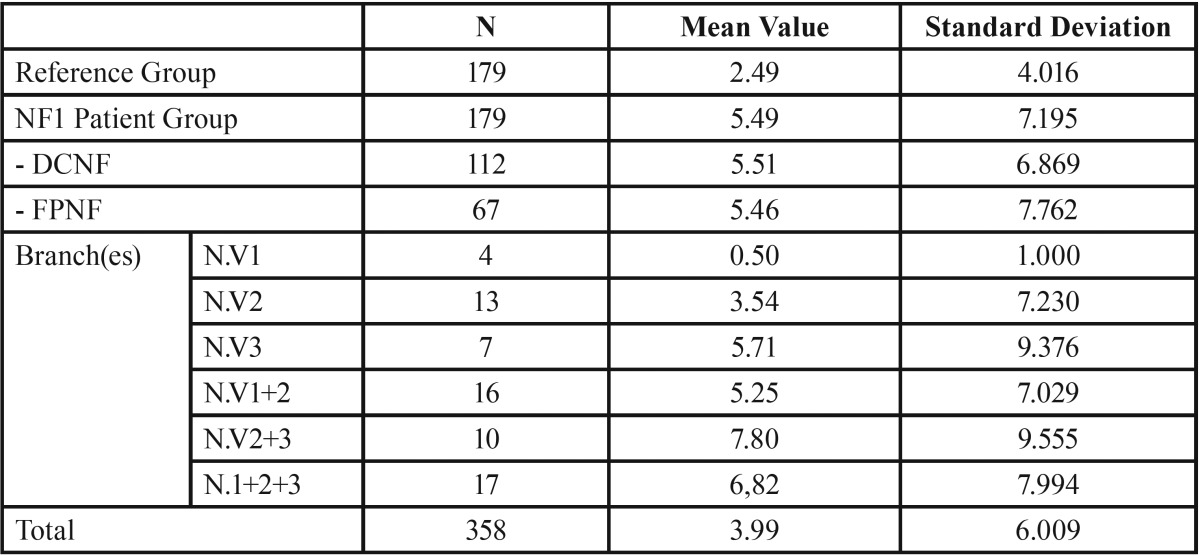
Number of missing teeth in reference group and patient group. Patient group is further specified for trigeminal nerve branch affected by facial plexiform neurofibroma (DCNF = disseminated cutaneous neurofibroma; FPNF = facial plexiform neurofibroma; N. V = nervus trigeminus; numbers 1 and 3 refer to trigeminal nerve branch(es) affected by PNF: 1 = ophthalmic branch, 2 = maxillary branch, 3 = mandibular branch).

The next investigation focused on the side-specific impact of FPNF on the number of missing teeth. Fourteen teeth were considered on each body side. Intra-individual comparison of the number of missing teeth with respect to localization of FPNF revealed statistically significant differences of mean values (FPNF, affected side: 3.01; FPNF, non-affected side: 2.42, *p* < 0.013). However, the distribution of missing teeth in the FPNF group needs further specification. More than half of the FPNF patients (34/67) showed an equal number of missing teeth on both sides of the jaws. In 11 other patients, the number of missing teeth was higher on the side not affected with a PNF. However, in these cases the number of missing teeth never exceeded two. In 22 cases the number of missing teeth was substantially higher on the affected side and could increase up to 11 missing teeth ( Table 6). The comparison of measured values showed how frequently more teeth were missing on either the tumorous side (positive sign) or the non-affected side (negative sign). A value of “0” indicates an equal number of lost teeth, taking into account the maximum number of 14 teeth per two jaw sides ( Table 6- Table 8).

**Table 6:**
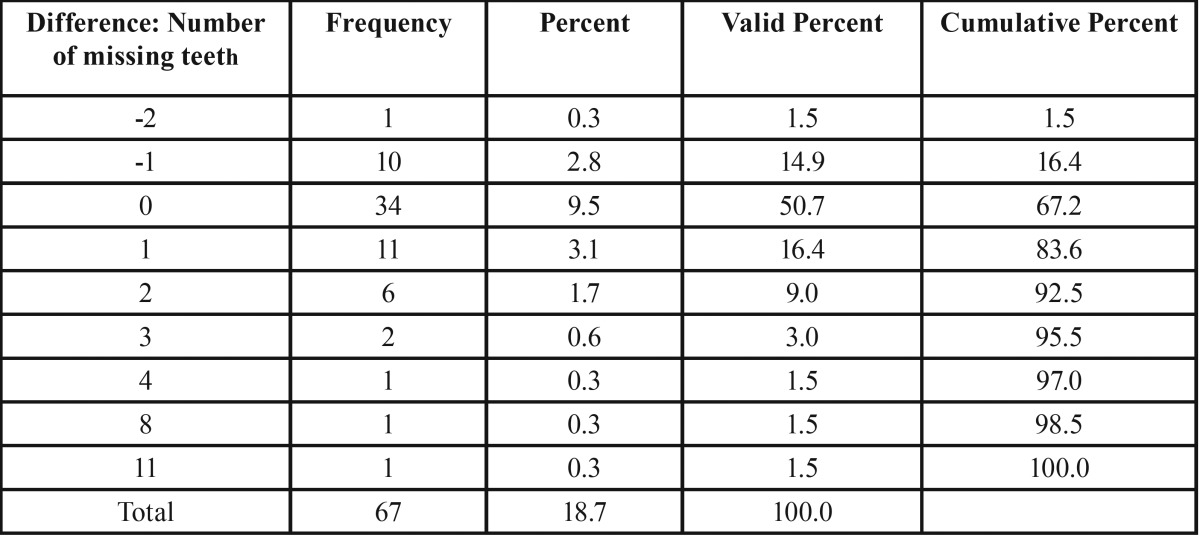
Differences between number of missing teeth per body side with respect to the side affected by facial plexiform neurofibroma (FPNF group, n = 67). Nearly half of patients show no difference concerning this item. In many patients, substantially more teeth are missing on the tumorous side.

**Table 7:**
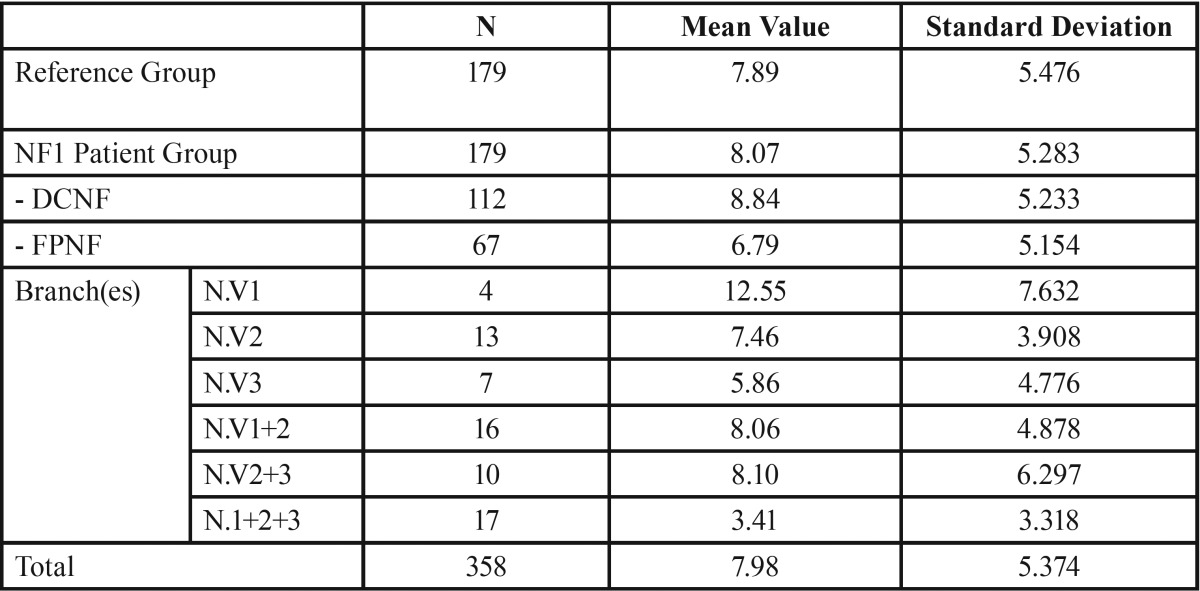
Number of filled teeth in reference group and patient group. Patient group is further specified for trigeminal nerve branch affected by facial plexiform neurofibroma (DCNF = disseminated cutaneous neurofibroma; FPNF = facial plexiform neurofibroma; N. V = nervus trigeminus; numbers 1 and 2 refer to trigeminal nerve branch affected by PNF: 1 = ophthalmic branch, 2 = maxillary branch, 3 = mandibular branch).

**Table 8:**
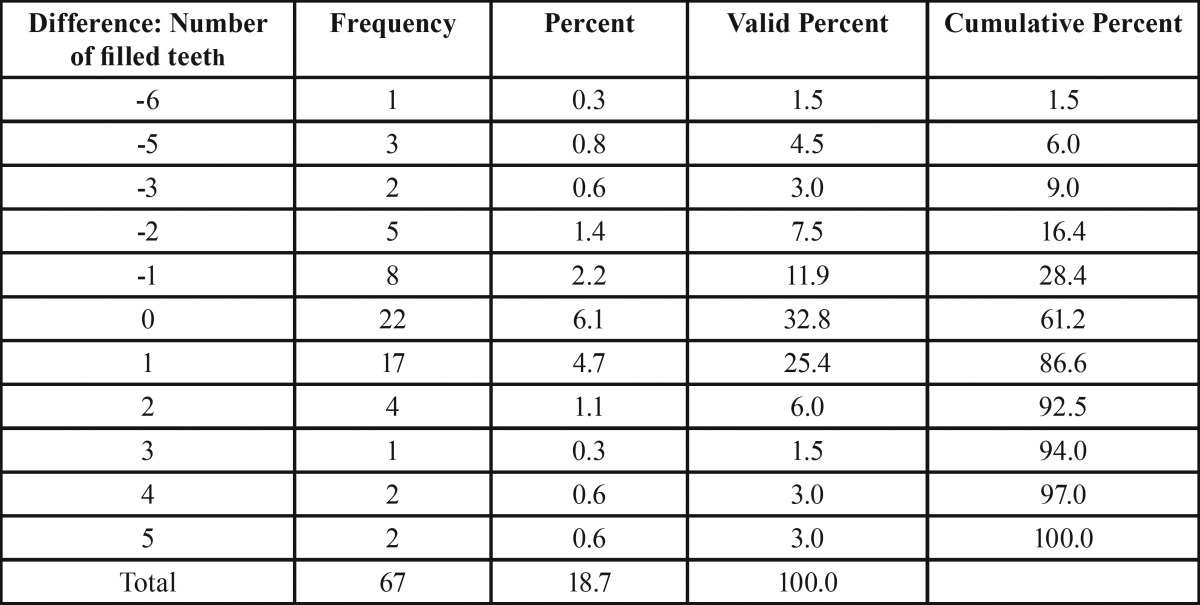
Differences between the number of filled teeth with respect to the side affected by facial plexiform neurofibroma (FPNF group, n = 67).

Restored teeth. The mean number of filled teeth was almost equal in both groups (reference group: 7.89; NF1 group: 8.07, *p* = 0.746). Interestingly, the mean value of dental fillings was higher in the DCNF group (8.84) than the FPNF group (6.79). The reason for this difference is the high number of FPNF patients with hemifacial tumor spread. In this group, the number of filled teeth was very low (3.41). On the other hand, FPNF patients affected in the ophthalmic branch only showed by far the highest values of filled teeth (12.25). However, this group of patients was small (n = 4).

**Figure 1:**
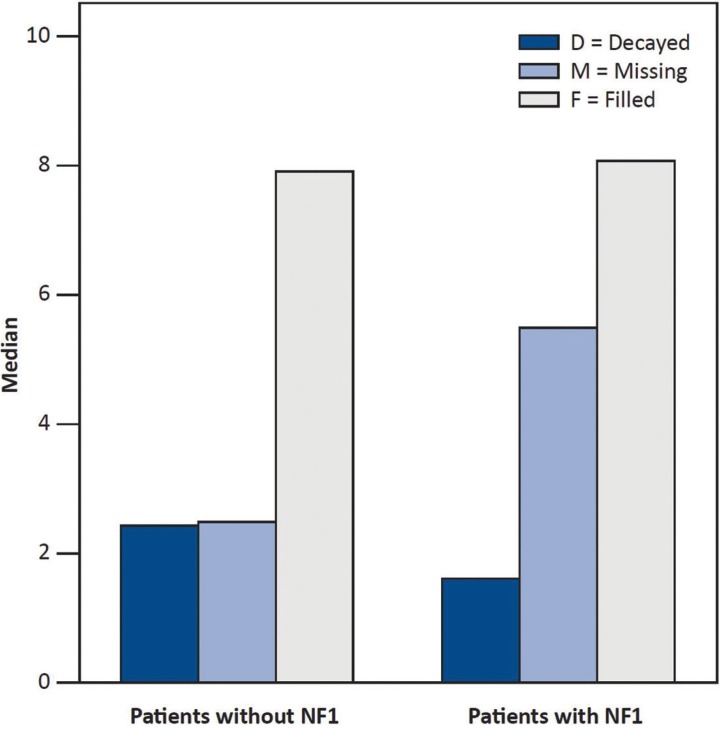
Illustration showing median values of parameters constituting the DMFT index.

Intra-individual comparison of the number of filled teeth with respect to FPNF localization showed no substantial difference (FPNF side: 3.42; non-FPNF side: 3.40). Figure 1 illustrates the proportions of the median values that make up the DMFT index. Particularly noteworthy is the distinct difference in missing teeth between the dentitions of the reference group and NF1 patients.

## Discussion

This study reveals an impact of the tumor predisposition syndrome NF1 on essential parameters of dental health. Although it is a retrospective evaluation of radiographs and the causes of the findings cannot be derived from imaging alone, the influence of the disease on the parameters of number, caries, and filling of teeth becomes clear. In addition, the tumor type is particularly important for the measured values.

The results can be compared with a recent report on a German oral health study report (Deutsche Mundgesundheitsstudie [DMS] IV) (32). According to DMS IV, the DMFT index in adults aged 35 to 44 years is 14.5. In comparison, the DMFT index of NF1 patients is higher (15.15) and of the reference group lower (12.82) than the mean value of the investigated people of the German Federal Republic. However, with regard to the age structure of this study, it is important to note that even younger individuals were included in our study. This difference may explain why the DMFT index of the reference group is below the value of the DMS IV study. On the other hand, from this emphasis on the different age structures of the two studies, it follows that the higher DMFT value of the patient group is even more significant, because significantly more young patients were included in the present evaluation than in DMS IV. Furthermore, the data of DMS IV rely on clinical investigations, but the present study relies on the analysis of radiographs only. Both methods have pros and cons (e.g. occlusal caries are more easily diagnosed clinically, whereas diagnosis of proximal caries is in the field of dental radiology). These methodological differences may contribute to different results.

Single factors of the DMFT index can be compared with the DMS IV results. The mean number of missing teeth in adults in Germany is 2.4 (31). It follows that the number of missing teeth in NF1 patients (5.49) exceeds this comparison by more than double. The mean value of decayed teeth is 0.5 in DMS IV. This value is three times higher for NF1 patients with 1.59. However, the number of decayed teeth also is high in the reference group of this study. An explanation for this difference in the expected oral health status is likely the referral characteristics for a university dental clinic. Patients with low income can receive low-cost dental care in training courses of dentistry students. Furthermore, this university dental clinic is the only one in this federal state and therefore an important source of care for the disabled. However, the mean value of filled teeth in Germany is 11.7 and thus higher than in NF1 patients (8.07).

The number of missing teeth is higher and of filled teeth lower in NF1 patients compared to the reference group (DMS IV). These results suggest that less filled teeth are to be calculated because there are fewer teeth in this group. This study did not clarify why more teeth were lost or extracted in NF1 patients compared to the reference group. This fact applies to both groups of NF1 patients. It is particularly interesting to note that in the group with hemifacial neurofibroma, significantly more teeth were absent on the affected jaw side. It would be interesting to investigate whether these tooth losses accumulated due to carious destruction. An obvious assumption that may be discussed here could be an increased caries risk due to the sometimes very pronounced tooth misalignments with associated jaw deformities in patients with FPNF.

In these cases, the oral hygiene may be considerably impaired, not least because of the tumorous destruction of masticatory muscles and poor function of the facial nerve that frequently occur on the FPNF-affected side. Another speculative factor of premature tooth loss could be the often difficult dental care of these patients with pronounced tooth misalignment. In addition to these morphological disabilities, there is considerable psychological strain on these syndrome patients, who often complain about distortions and social deprivation (33). The influence of these factors on dental health is not included in this study.

Data on dentition are sparse in NF1 patients (34). In particular, only few data are available on the frequency and causes of numerical aberrations of permanent teeth in NF1 (22), in particular genetically caused changes in the number of teeth. Hypodontia of the NF1 group must be considered as an explanation for the differences between the two groups (22). However, our clinical data are based on a cross-sectional study and thus are incomplete to address this biographic parameter. A large body of literature exists with descriptive reports of dental anomalies and mandibular deformities. These findings can be attributed to the variable form of FPNF-associated bone deformations in the vast majority of cases (18,19,22,23). With reference to dental findings, these reports detail the numerous retentions of teeth but do not address the potential impact of the disease on formation of teeth and dental health. Nevertheless, a radiographic cross-sectional study is superior to a study relying on oral inspection (25). In particular, complex retentions of teeth have to be expected in NF1 (25).

Two oral health studies on NF1 patients have already been performed in other countries.

A study from Canada (27) revealed significantly higher rates of decayed teeth in patients with NF1 than in a reference group of healthy individuals. This study was carried out on the basis of a questionnaire sent to families of which at least one member was suffering from NF1. The authors pointed out that caries were much more common in the affected NF1 patient than in the other members of the family. However, the meaningfulness of a questionnaire, which is based on dental findings collected by lay persons themselves, is obviously limited.

A Finnish study on dental health was performed on a large group of NF1 patients. In this study, 110 patients with NF1 aged 3 to 68 years were clinically and radiologically examined for carious, missing, and filled teeth. The results were summarized in age cohorts and compared with the results of large national studies on dental health (28). In the age groups of the under 20-year-old NF1 patients, it was examined what percentage of students had a DMFT index of more than zero. Contrary to the expectations of the investigators, the proportions of treated teeth in the NF1 patients in all age classes of the under 20-year-olds were significantly lower than those of the reference groups. The proportion of a DMFT > 0 was between 0% and 21% in the NF1 group, with the values of the reference group between 16% and 84%. On the other hand, the DMFT values were similar for the older age groups between the two groups. As a possible reason for the results of the study, it was discussed that participants in an oral health study per se have a higher interest in their own dental health, and therefore the results could be biased. Furthermore, growing knowledge about the NF1 disease could have led to the patients’ more careful handling of their own body. This change in consciousness could also be of significance to one’s own oral hygiene, have positively influenced the education of affected children, and have also reached the treating dentists through public relations work (28).

Comparing the Finnish results with our own findings, the factor of volunteering in the examination of the dental health of NF1 patients falls away, because this was a routine examination in the framework of the dental control of an outpatient clinic specialized in treatment of NF1 patients. On the other hand, the findings are likely to reflect the spectrum of dental health of NF1 patients in Germany, as patients from all federal states are recruiting. Our results are in line with the Canadian report on the dental health of NF1 patients, as both studies evidently show a lower rate of oral health in patients compared to the control group (27). Currently, there is no adapted dental health program that may meet the higher needs of education in this patient group (35).

## Conclusions

NF1 is recognized worldwide as a human tumor and malformation syndrome with no ethnic preference. The comparison of our data with the results of the studies from Canada and Finland on dental health in NF1 patients adds the new finding that facial tumor type should be considered in the evaluations of oral health in NF1-affected individuals.
